# Unobtrusive Estimation of Cardiac Contractility and Stroke Volume Changes Using Ballistocardiogram Measurements on a High Bandwidth Force Plate

**DOI:** 10.3390/s16060787

**Published:** 2016-05-28

**Authors:** Hazar Ashouri, Lara Orlandic, Omer T. Inan

**Affiliations:** School of Electrical and Computer Engineering, Georgia Institute of Technology, Atlanta, GA 30332, USA; lorlandic3@gatech.edu (L.O.); inan@gatech.edu (O.T.I.)

**Keywords:** ballistocardiography (BCG), heart failure, unobtrusive cardiovascular monitoring, stroke volume, cardiac contractility

## Abstract

Unobtrusive and inexpensive technologies for monitoring the cardiovascular health of heart failure (HF) patients outside the clinic can potentially improve their continuity of care by enabling therapies to be adjusted dynamically based on the changing needs of the patients. Specifically, cardiac contractility and stroke volume (SV) are two key aspects of cardiovascular health that change significantly for HF patients as their condition worsens, yet these parameters are typically measured only in hospital/clinical settings, or with implantable sensors. In this work, we demonstrate accurate measurement of cardiac contractility (based on pre-ejection period, PEP, timings) and SV changes in subjects using ballistocardiogram (BCG) signals detected via a high bandwidth force plate. The measurement is unobtrusive, as it simply requires the subject to stand still on the force plate while holding electrodes in the hands for simultaneous electrocardiogram (ECG) detection. Specifically, we aimed to assess whether the high bandwidth force plate can provide accuracy beyond what is achieved using modified weighing scales we have developed in prior studies, based on timing intervals, as well as signal-to-noise ratio (SNR) estimates. Our results indicate that the force plate BCG measurement provides more accurate timing information and allows for better estimation of PEP than the scale BCG (*r*^2^ = 0.85 *vs.* *r*^2^ = 0.81) during resting conditions. This correlation is stronger during recovery after exercise due to more significant changes in PEP (*r*^2^ = 0.92). The improvement in accuracy can be attributed to the wider bandwidth of the force plate. ∆SV (*i.e.*, changes in stroke volume) estimations from the force plate BCG resulted in an average error percentage of 5.3% with a standard deviation of ±4.2% across all subjects. Finally, SNR calculations showed slightly better SNR in the force plate measurements among all subjects but the small difference confirmed that SNR is limited by motion artifacts rather than instrumentation.

## 1. Introduction

Cardiovascular diseases (CVD) are the leading cause of death in the United States, according to the American Heart Association. A particularly prevalent CVD is Heart Failure (HF), which affects 5.7 million Americans and causes one in nine national deaths [1]. HF is characterized by the heart’s inability to provide sufficient blood to the organs and tissues [2]. The annual health care costs of HF in the United States are $30.7 billion, most of which relate to hospitalization costs [3].

Readmission rates to the hospital after a discharge for HF patients are 25% within 30 days. Often, readmissions result from cardiovascular events, such as when ejection fraction is insufficient, and ventricular filling pressures are subsequently elevated; these elevated pressures are observed at least 6 days prior to the onset of symptoms such as shortness of breath or edema [4]. Studies have shown that out-of-clinic monitoring of HF patients can (1) reduce hospital and emergency room visits; (2) reduce healthcare costs associated with the disorder; and (3) lead to a decrease in mortality [5,6,7]. Although the CardioMEMS Implantable Hemodynamic Monitor (IHM) has recently received U.S. Food and Drug Administration (FDA) approval for this purpose of enabling home monitoring of HF patients, the cost per patient of implantation exceeds $25,000. Hence, providing a noninvasive, unobtrusive, and inexpensive alternative for outside-of-clinic monitoring of HF patients is imperative to diminishing the financial burden of the disease and improving the patients’ quality of life [8].

HF is associated with a weakened myocardium with impaired contractility. The time elapsed between the electrical depolarization of the ventricular muscle and the ensuing opening of the aortic valve has been shown to be a surrogate for cardiac contractility that can be measured noninvasively as the interval between the electrocardiogram (ECG) Q-wave and the opening of the aortic valve [9]. Specifically, an increased pre-ejection period (PEP) is an indication of decreased contractility [10]. Moreover, because the heart cannot contract efficiently, stroke volume (SV), which is the volume of blood ejected by the left ventricle in one contraction, decreases in HF patients prior to exacerbation. Therefore, measuring a patient’s PEP and SV changes outside the clinic can provide insight into the severity of his/her condition and can potentially allow prediction of exacerbations, and physicians to intervene and amend medications to prevent such exacerbations and hospitalizations [11]. Currently, one reference standard noninvasive means of measuring PEP and SV is impedance cardiography (ICG) [12]. However, ICG is impractical in an out-of-clinic setting as it requires a trained medical professional to apply eight electrodes to the subject and operate the measuring device [13]. In order to empower HF patients to monitor their condition outside the clinic, there needs to be an affordable, unobtrusive method for measuring PEP and SV changes.

Ballistocardiography (BCG), a measurement of the recoil forces of the body in response to the ejection of blood from the heart and movement of the blood through the vasculature [14,15] has been demonstrated as an alternative to ICG for measuring PEP [13]. In that prior study, BCG signals were measured using a modified electronic weighing scale, and we showed that this scale could be modeled as a second-order mechanical system. We characterized the spring constant and associated mechanical bandwidth for a range of bodyweights for typical adults (>15 Hz bandwidth for bodyweights of 150 kg or less) [16]. This mechanical bandwidth, while sufficient for not attenuating any of the frequency components of interest for BCG recordings, may potentially distort the BCG waveform and reduce the accuracy of timing interval measurements due to uneven group delay in the pass-band. Accordingly, in this paper, we compare the modified weighing scale to a high bandwidth, reference standard force plate to evaluate the accuracy in PEP estimation. We also estimate per subject ∆SV (*i.e.*, relative changes in SV) using the force plate BCG and verify the accuracy against simultaneous ICG measurements. Finally, we provide signal-to-noise ratio (SNR) calculations of scale-based BCG and force plate BCG measurements to understand whether the higher resolution of the force plate is beneficial.

## 2. Methods and Materials

### 2.1. Protocol

The study was conducted under a protocol reviewed and approved by the Georgia Institute of Technology Institutional Review Board (IRB). All subjects provided written consent before experimentation. Data were collected from 17 healthy subjects (Gender: 10 males, 7 females; Age: 23.6 ± 4.5 years, Height: 172.8 ± 9.9 cm, Weight: 70.7 ± 11.3 kg). Each subject was asked to stand still in an upright position for 60 s on each of the force plate and scale for baseline measurements. After that, each subject performed a stepping exercise for 60 s after which they were asked to stand on the scale as still as possible for 5 min to monitor their full recovery. Half of the subjects stood on the scale first during baseline measurement while the other half stood on the force plate first to account for any bias due to the order in which the subject stood on the force plate *versus* the scale. In addition to the BCG, we measured the ECG and ICG. The ECG was used as timing reference to ensemble average the BCG and ICG signals. The B-point of the ICG was used as a reference method for detection of the opening of the aortic valve [17,18]; it was also used for reference ∆SV calculations.

### 2.2. Hardware Design

**Figure 1:**
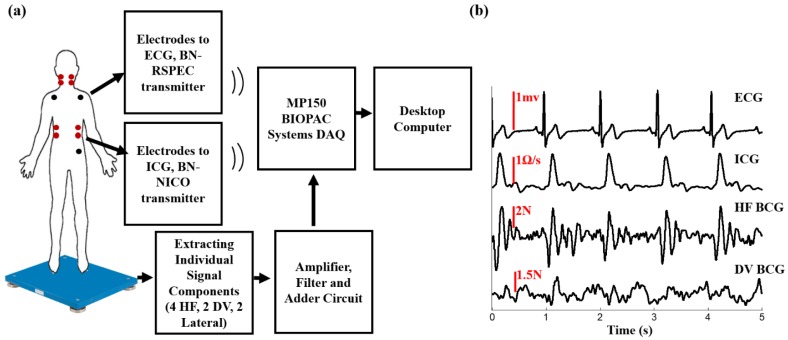
(**a**) A block diagram of the experimental setup; (**b**) a 5 s time trace showing ECG, $\frac{d}{\mathrm{dt}}$ ICG, head-to-foot force plate BCG.

Two different instruments were used to measure the BCG; a modified electronic weighing scale and a high bandwidth multi-component force plate. The modified weighing scale (BC534, Tanita, Tokyo, Japan) was developed in previous work using an analog amplifier and a strain gauge bridge [16]. The multi-component force plate (Type 9260AA6, Kistler Instrument Corp, NY, USA) has a bandwidth in excess of 200 Hz and sufficient resolution to enable BCG measurements in all three axes. The force plate has four, three-component force sensors. Each of the four sensors has three pairs of quartz plates, one sensitive to pressure in the z-direction (head-to-foot) and two to shear in the x and y-directions (dorsoventral and lateral respectively). Out of the 12 output signals, two of the shear forces that have the same line of action can be paralleled so the outputs of the force plate is eight signals instead of 12. Since we only process head-to-foot BCG in this work, we accessed each of the 4 signals in the z-direction separately, passed each one of them as an input to an amplifier and filter circuit and then the outputs of these four components were added using an adder circuit. The summed, amplified and filtered head-to-foot BCG signal was outputted into the data acquisition system (MP150, BIOPAC System Inc., Goleta, CA, USA). The ECG and ICG signals were measured using BN-RSPEC and BN-NICO wireless modules (BIOPAC Systems, Inc., Goleta, CA, USA), respectively, then transmitted to the MP150. All signals were sampled at 2 kHz. Figure 1a,b shows a block diagram of the experimental setup and a time trace of ECG, $\frac{d}{\mathrm{dt}}$ ICG, and head-to foot BCG.

### 2.3. Data Processing

The BCG, ICG, and ECG signals were filtered with finite impulse response (FIR) Kaiser window band-pass filters with cut-off frequencies as follows: 0.9–30 for ICG and ECG, 0.5–20 for head-to-foot BCG. The ECG R-peaks were detected using an automated peak detection algorithm and verified manually. This was done by computing a constant threshold of 50% of the maximum amplitude of the absolute value of the band-pass filtered ECG signal for each subject; local maxima greater in amplitude than this threshold were then located automatically and annotated as R-waves. The minimum R-R interval was calculated for every subject and the detected R-peaks were used as a fiduciary to segment ICG and BCG signals into individual heart beats each with a window length equal to the minimum R-R interval. These extracted heartbeats were then averaged to obtain ensemble averaged traces with reduced noise.

### 2.4. Feature Extraction and Statistical Analysis

**Figure 2:**
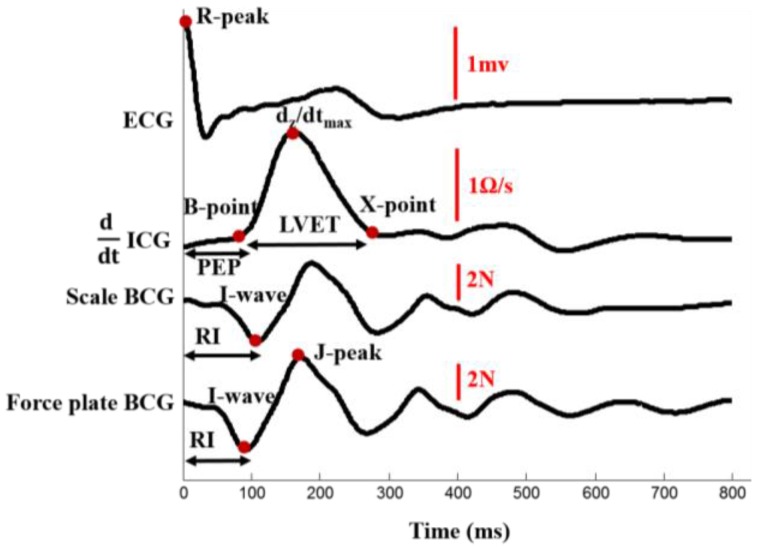
Ensemble averaged traces of ECG, $\frac{d}{dt}$ ICG, scale BCG, and head-to-foot force plate BCG with the characteristic points and features.

The I-wave in the weighing scale and force plate BCG signals was obtained by detecting the minima before the global maxima in the first 200 ms portion of the corresponding BCG ensemble average. The J-peak and its amplitude (from the reference threshold, *i.e.*, 0) were detected as the global maxima of the force plate and scale BCG ensemble averages. The B-point of the first derivative of ICG was detected as the global maxima of the second derivative of the ICG [13]. The X-point was obtained by detecting the minima after the global maxima of the first derivative of ICG. The amplitude of the maxima of the first derivative of ICG was also detected. Figure 2 shows ensemble averages of ECG, $\frac{d}{\mathrm{dt}}$ ICG, scale BCG and head-to-foot force plate BCG signals with the extracted features.

#### 2.4.1. Correlation at Rest

The 60 s baseline recording of scale BCG, force plate head-to-foot BCG, and ICG were divided into 12 sub-ensembles (5 s long) and a sub-ensemble average was obtained for each of the 12 sub-ensembles. For each of those sub-ensembles, the RI duration was calculated (the timing at which the I-wave occurs since the ensemble averaging is done with respect to the ECG R-peak) and PEP was calculated (the timing at which the B-point—opening of the aortic valve—occurs). Although PEP is defined as the time elapsed between the Q-point in the ECG and the Bpoint in the ICG, detecting the Q-point in the ECG is not always as robust as detecting the R-point. Hence, in our analysis we used the R-peak in ECG as a reference and estimated PEP as the RB-interval since we are most interested in relative changes in PEP rather than absolute measures, and the QR interval is typically consistent from beat-to-beat A correlation analysis was then performed between RI-intervals in both scale and force plate and the corresponding PEP values (from ICG) among all 17 subjects.

#### 2.4.2. Correlation during Exercise Recovery

The 5 min after exercise recovery recording of head-to-foot force plate BCG and ICG were divided into 15 sub ensembles (20 s long). As compared to the rest data, a longer time was required for extracting the sub-ensemble averages (20 s compared to 5 s) due to increased postural sway in exercise recovery compared to rest. A sub-ensemble average and RI-intervals and PEP were calculated for each of the 15 sub-ensembles. A correlation analysis was then performed between RI-intervals and PEP among all 17 subjects.

### 2.5. Estimating Relative Changes in Stroke Volume

Stroke volume SV1 was calculated for each subject during rest using the reference ICG signal using Sramek’s equation [19]:

(1) $$SV1=\frac{{\left(0.17H\right)}^{3}}{4.25Z_{0}}\cdot \left(\frac{d_{z}}{dt_{\max}}\right)\cdot LVET$$

where H is the height of the subject, $Z_{0}$ is the base impedance of ICG, $d_{z}/dt_{max}$ is the amplitude of the global maxima of the first derivate of ICG, and LVET is the left ventricular ejection time which is calculated as the timing difference between X-point and B-point in the first derivative of ICG.

Then, for each of the 17 subjects, separately, the 5 min after exercise recovery recording of head-to-foot force plate BCG and ICG were divided into 15 20-s sub-ensemble averages (as with the PEP estimation described above). The normalized change in stroke volume $\Delta SV_{i}$ for each of the 15 sub-ensemble averages was calculated as follows:

(2) $$\Delta SV_{i}=\frac{SV_{i}-SV1}{SV1}$$

For each subject, subject specific correlations were performed between the J-peak of the force plate BCG and the X-point of the first derivative of ICG and for a linear regression model was calculated such that:

(3) $${\overset{⌢}{X}}_{i}=\alpha_{1}\times Jpeak_{i}+\alpha_{2}$$

where ${\hat{X}}_{i}$ is the estimated X-point for sub-ensemble average i. Similarly, subject specific correlations were performed between the I-wave of the force plate BGC and the B-point of the first derivative of ICG and for every subject a linear regression model was calculated such that:

(4) $${\overset{⌢}{B}}_{i}=\beta_{1}\times Iwave_{i}+\beta_{2}$$

where ${\hat{B}}_{i}$ is the estimated B-point for sub-ensemble average i. Estimated LVET for each of the 15 sub-ensemble averages for each subject was then calculated as:

(5) $${\hat{LVET}}_{i}={\hat{X}}_{i}-{\hat{B}}_{i}$$

Similarly, another subject-specific correlation was performed between the amplitude of the J-peak and $d_{z}/{\mathrm{dt}}_{\max}$ and the linear regression model was used to estimate $d_{z}/{\mathrm{dt}}_{\max}$ for the sub-ensemble averages for that subject. Finally, the estimated ∆SV was calculated for each of the 15 sub-ensemble averages for every subject and the resulting estimates were compared with the reference calculated changes in stroke volume.

### 2.6. Signal-to-Noise Ratio Calculations

For each of the 17 subjects, an ensemble average $\bar{x}$ was obtained for the 60 s baseline recording of weighing scale BCG and head-to-foot force plate BCG. The 60 s window was then divided into 12 sub-ensembles (5 s long) $x_{i}$ and a sub-ensemble average ${\bar{x}}_{i}$ was obtained for each of the 12 sub-ensembles. Five-second ensembles were used rather than individual beats to reduce motion artifacts. Noise was calculated for each of the sub-ensemble averages using:

(6) $$n_{i}={\bar{x}}_{i}-a_{i}\bar{x}$$

where $a_{i}$ is the normalization coefficient for the amplitude of the ensemble averaged BCG w.r.t the ith sub-ensemble average:

(7) $$a_{i}=\frac{{\bar{x}}_{i}\cdot \bar{x}}{\bar{x}\cdot \bar{x}}$$

The noise-to-signal ratio (NSR) was then calculated for each of the sub-ensemble averages:

(8) $$NSR_{i}=\frac{\mathrm{var}(n_{i})}{\mathrm{var}(\bar{x})}$$

Finally, SNR was calculated as follows:

(9) $$SNR=\frac{1}{\frac{1}{12}\sum_{i=1}^{12}NSR_{i}}$$

## 3. Results and Discussion

### 3.1. RI and PEP Correlation for Scale and Force Plate BCG during Rest

**Figure 3:**
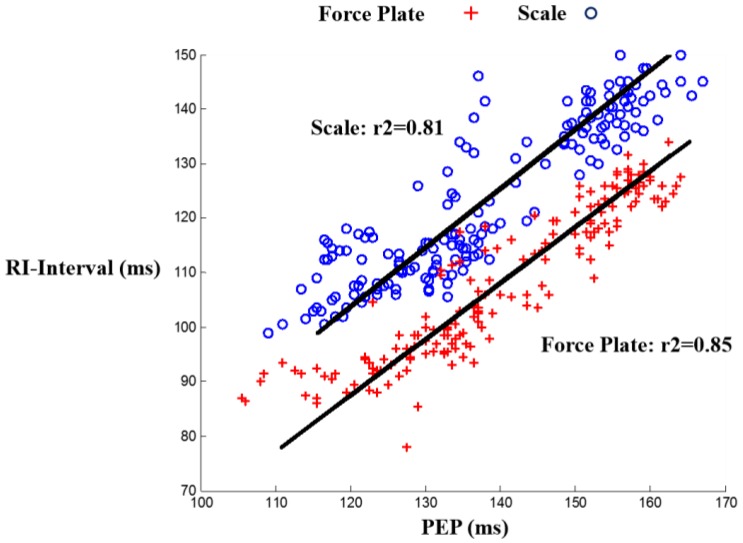
Linear regression fit for both scale and force plate head-to-foot BCG RI-interval *vs.* PEP among all subjects.

**Figure 4:**
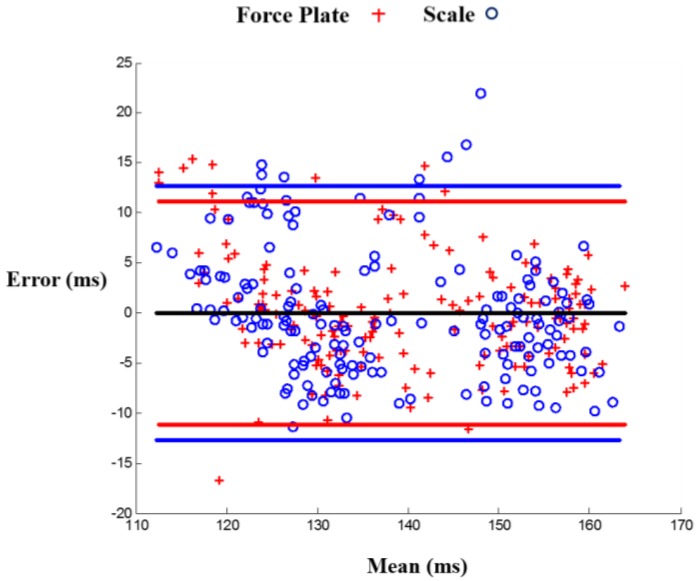
Bland Altman plot for scale and force plate linear prediction models of PEP. The blue line is the 95% confidence range of the PEP estimations from the scale BCG RI-interval while the red line is the 95% confidence range of the PEP estimations from the force plate BCG RI-interval.

The correlation results were: $r^{2}=0.85$, m=0.97, b=34.92 for the force plate and $r^{2}=0.81$, m=0.92, b=24.23 for the scale, with *m* being the slope and *b* being the y-intercept. These results show an improved timing accuracy in force plate compared to scale BCG in estimating PEP, demonstrating that the limited mechanical bandwidth in the weighing scale impacts the timing accuracy. Figure 3 shows a linear regression fit for scale and force plate BCG RI-intervals *vs.* PEP, and Figure 4 is a Bland Altman plot [20] that shows a larger standard deviation in PEP estimated from scale BCG compared to PEP estimated from force plate BCG.

### 3.2. RI and PEP Correlation for Head-to-Foot Force Plate BCG during Recovery

**Figure 5:**
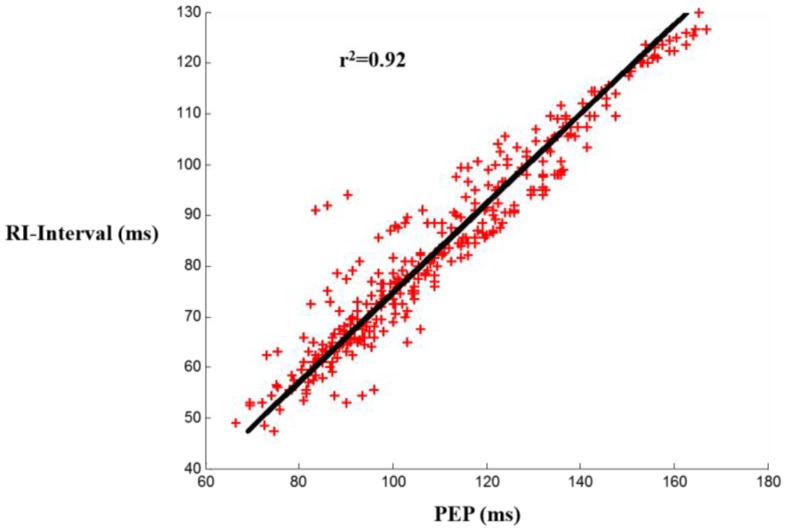
Linear regression fit for head-to-foot force plate BCG *vs.* PEP during recovery among all 17 subjects.

The results were: $r^{2}=0.92$, m=1.14, b=14.64. Figure 5 shows a linear regression fit for force plate head-to-foot BCG *vs.* PEP during recovery. The higher correlation of head-to-foot BCG I-wave to PEP during exercise recovery compared to that during rest ($r^{2}=0.85$) can be attributed to the more significant changes in PEP and RI intervals during exercise recovery as both values increase to stabilize again at the baseline values. These results show a better correlation coefficient (0.92 *versus* 0.86) and a much smaller y-intercept (14.6 *versus* 138) than the results obtained from correlating RJ interval to PEP in our previous work [16]. One of the future work objectives in that paper was to examine waves other than the J-wave for better correlation and smaller y-intercept but that was not feasible because of the difficulty of detecting those other waves during exercise recovery. However, with the force plate BCG, we were able to successfully extract the I-waves from all subjects and hence obtain the improved results. 

### 3.3. Stroke Volume Estimation from Head-to-Foot Force Plate BCG during Recovery

**Figure 6:**
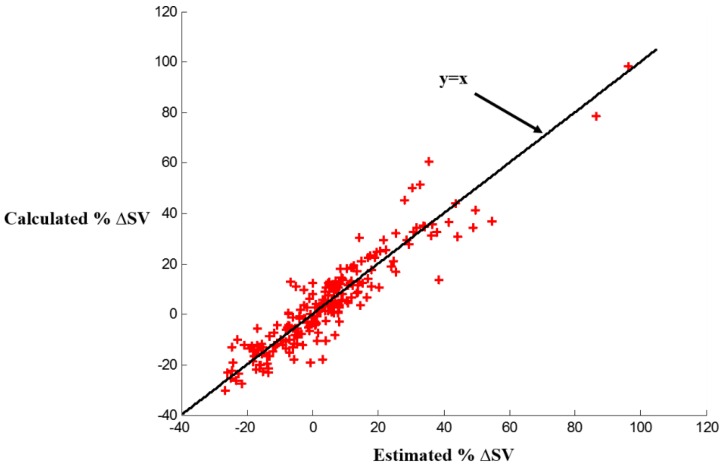
Estimated stroke volume percent changes from head-to-foot force plate BCG compared to calculated stroke volume percent changes from reference ICG.

The mean error among all subject was 5.3% with a standard deviation of 4.2%. We compared our results to other studies providing such detailed error analysis information on non-invasive SV estimation. In the ones available [21,22,23,24], the following results were achieved and considered “acceptable”. In [21], the mean error was 16.5% for SV derived from femoral arterial blood pressure (ABP) and 14.5% for SV derived from radial ABP. In [22], the mean error was 8.9% for SV estimated using mean arterial pressure (MAP) and 14.35% for SV estimated using the systolic waveform. In [23], the percentage error between electric velocimetry and transesophageal Doppler echocardiograph for estimating cardiac output (CO)—which is obtained from multiplying SV by heart rate—was 29%. All of these results were considered acceptable. In [24], the comparison between SV estimation using the Vigileo-FloTrac™ system and esophageal Doppler yielded an error rate of 58% which was deemed unacceptable, since according to [25], limits of agreement of ±30% are acceptable. Hence, our method allows SV changes estimation for every subject accurately and can provide insight in the case of HF patients. The per subject results of the absolute and percentage mean and standard deviations of the estimated changes in stroke volume compared to the reference calculations are shown in Table 1. Figure 6 also shows the percentage estimated stroke volume changes in comparison with the calculated percentage stroke volume changes from ICG. We can observe in Figure 6 that the increase in SV positive in some cases while being negative in other cases. This can be attributed to the fact that SV increases during exercise and then decreases again during exercise recovery and it sometimes goes below the baseline SV by the end of the recovery period. Hence, at the beginning of the recovery period, the change in SV would be positive while at the end of the recovery period the change in SV would be around zero or sometimes negative.

### 3.4. Signal-to-Noise Ratio Comparison for Scale BCG and Force Plate Head-to-Foot BCG

SNR results show slightly better SNR values for force plate head-to-foot BCG compared to scale BCG. The results for SNR calculations are shown in Table 2.

The force of the BCG signal is 2 Npp and the sensitivity of the transducer in the weighing scale is 19.1 μV/N [16] which would give a BCG voltage of 38.2 μVpp. The noise level referred to the input of the circuit is 150 nVpp. This would lead to an SNR of 255 in the linear scale or 48 dB. As for the force plate, with the same BCG amplitude of 2 Npp for the BCG and a sensitivity of 19 mV/N amplified by 250 times in the z-direction gives a signal of 9.5 Vpp. The output noise level is 5 mVpp which leads to an SNR of 1900 in the linear scale or 66 dB. Those calculations are clearly far better from what we are obtaining in terms of SNR for both the scale and force plate so the difference cannot be attributed to this. Hence, it is likely that the BCG SNR is limited by motion artifacts, not electronic noise. 

We attribute the improvement in SNR in the force plate BCG over scale BCG to the fact that a subject can stand more comfortably on the force plate with their feet wider apart than with the scale. To further investigate this theory, we had one subject stand on the force plate with feet 12.7 (same distance as for the scale), 20.3, 25.4 and 35.6 cm apart. The SNR results were as follows: 2.2, 2.9, 4.4, and 4.3 dB, respectively, suggesting that there is a slight improvement in SNR associated with allowing the feet to be wider apart as compared to the scale. This goes in accordance with Reference [26] which states that stance width influences the magnitude of body sway and observed that for subjects standing with their feet 5, 17, and 30 cm apart and exposed to visual motion, the widest distance (30 cm) was associated with reduced motion sickness. 

Moreover, we observed the order in which subjects stood on the scale and force plate (since half the subjects stood on scale first while the other half stood on the force plate first to account for any bias due to the order in which the subject stood on the force plate *versus* the weighing scale), and found that the difference in SNRs between force plate and weighing scale BCG was bigger for all the subjects that stood on the force plate first (subjects: 2, 3, 5, 6, 8, 10, 11, and 12 in Table 2) compared to those who stood on the weighing scale first (subjects: 1, 4, 7, 9, 13, 14, 15, 16, and 17 in Table 2). For both groups, the results are statistically significant (*p* < 0.05). 

### 3.5. Limitations

One limitation of the work is that all participants are healthy, young subjects. Future work will focus on acquiring and analyzing data from a more diverse population and from HF patients in particular. The methods described in this paper set the technological framework for studies on HF patients. The main challenges in such patients are irregularities in the ECG signal whose R-peaks we use as reference to ensemble average the rest of the signals, as well as reduced signal quality in the BCG signals themselves.

## 4. Conclusions

We have shown in this paper that force plate BCG allows for more reliable PEP estimations than scale BCG due to more accurate timing information that can be attributed to the wider bandwidth of the force plate. Nevertheless, the cost of the force plate is significantly higher than the weighing scale, and thus the force plate—at this current cost—would not be feasible for at-home use. If the force plate was positioned in a central location accessible to multiple HF patients such as in a grocery store, the cost of the force plate could be amortized over the large number of patients that could access the device, thus allowing it to be a feasible option for monitoring patients outside the clinic/hospital setting. We have also shown that force plate head-to-foot BCG can be used to estimate SV changes accurately. However, in order to determine subject-specific coefficients for how the BCG parameters relate to the ICG parameters, simultaneous ICG measurements are needed at first for calibration purposes. Hence, the estimation of SV changes hinges on having the initial reference measurement. Nevertheless, for the HF patients monitoring application, such initial measurements of SV can be obtained at the index visit, and then the BCG-equipped force plate can be used to measure changes from that initial value over time. Changes in SV could then be used to titrate care, thus potentially leading to improved outcomes and reduced costs.

## Figures and Tables

**Table 1:** Per subject errors in ∆SV estimation.

Subject	µ Error (mL)	σ Error (mL)	% µ Error	% σ Error
1	2.0	1.7	3.6	3.1
2	1.4	0.9	3.4	2.3
3	3.7	2.6	6.5	4.6
4	4.0	4.9	5.4	6.7
5	2.5	1.8	6.4	4.7
6	2.8	2.0	8.9	6.4
7	3.4	3.1	6.6	6.0
8	2.8	2.6	4.6	4.3
9	5.6	4.9	11.0	9.5
10	3.4	2.5	4.5	3.4
11	3.1	3.0	5.3	5.1
12	3.3	1.4	5.2	2.1
13	1.6	1.3	3.3	2.8
14	1.9	1.4	4.6	3.3
15	1.0	0.8	2.2	1.8
16	3.4	1.8	4.7	2.5
17	3.5	2.5	4.6	3.2
Average	2.9	2.3	5.3	4.2

**Table 2:** Per subject SNR calculations for scale and force plate BCG.

Subject	Gender	Height (cm)	Weight (kg)	FP SNR (dB)	Scale SNR (dB)
1	Female	160	59	6.0	5.6
2	Male	175	75	8.6	7.6
3	Female	168	68	8.1	5.3
4	Female	160	52	1.7	1.3
5	Male	183	86	8.3	4.3
6	Male	175	74	1.5	-1.8
7	Female	152	49	5.5	4.6
8	Male	178	65	3.4	1.7
9	Male	178	88	1.6	1.5
10	Male	178	68	2.6	1.2
11	Male	190	88	7.1	5.1
12	Female	175	68	9.0	8.1
13	Male	175	70	3.8	3.8
14	Male	185	76	8.3	8.3
15	Male	175	79	3.1	2.7
16	Female	163	75	6.7	6.6
17	Female	168	61	8.0	7.8
